# A Rising Concern of Loperamide Abuse: A Case Report on Resulting Cardiac Complications

**DOI:** 10.7759/cureus.6314

**Published:** 2019-12-06

**Authors:** Amit Sapra, Priyanka Bhandari, Supriya Gupta, Shashank Kraleti, Ronak Bahuva

**Affiliations:** 1 Family and Community Medicine, Southern Illinois University School of Medicine, Springfield, USA; 2 Family and Preventive Medicine, University of Arkansas for Medical Sciences, Little Rock, USA; 3 Internal Medicine, Topiwala National Medical College, Mumbai, IND

**Keywords:** loperamide, prolonged qt interval, abuse, cardiotoxicity

## Abstract

The purpose of our case report is to generate awareness among the providers about the rising abuse of loperamide, which is a readily available nonprescription medication for its opiate-like actions and the risk of severe cardiac complications as a consequence of the same. It is currently becoming a significant concern among the healthcare fraternity due to its increasing abuse owing to its opioid agonistic activity. Our patient was a 32-year-old female who presented to the ED with ventricular dysrhythmias and persistent, prolonged QT interval secondary to excessively high doses of over-the-counter (OTC) loperamide abuse. More and more cases of loperamide abuse and its cardiotoxic potential are being reported in the literature, highlighting the increasing incidence of this problem.

## Introduction

Loperamide is an easily accessible over-the-counter (OTC) available anti-diarrheal medication, usually considered safe on a daily dose of 2-16 mg/day. Loperamide is a peripherally acting mu-opioid receptor agonist exerting its effects mainly on the myenteric plexus of the gastrointestinal longitudinal muscle layer and thus decreasing the propulsive activity of the intestine. At the recommended doses, it lacks any central nervous system (CNS) effects of euphoria and analgesia [1]. At high doses around 50 mg/day, however, it starts penetrating CNS and starts manifesting these central effects as well as cardiotoxic effects [2-6]. The recent few years have seen an upsurge in the loperamide abuse among the pain medication seeking population, coinciding with the increasing restrictions that started being placed on the prescription opioids. The abusers typically have been ingesting high doses of the medication in order to achieve or prolong their opioid agonistic action as well to alleviate the opiate withdrawal issues. It has been called the “poor man’s methadone” and is being increasingly used for the opiate withdrawal symptoms [2].

Interestingly enough, the increase in loperamide-related discussions in the fall of 2010 coincides with the introduction of reformulated, tamper-resistant oxycodone tablets [7]. The poison centers received more than twice the number of calls for intentional loperamide exposure between 2010 and 2015, and approximately 50% of abuse cases were reported after January 2014 [8]. Studies assessed the 2010-2015 US National Poison Data System loperamide exposures and identified a 91% increase of cases overtime with interestingly loperamide being the sole agent in 50% of the cases [9]. There is ample evidence of web-based discussions about loperamide’s use to self-treat withdrawal symptoms along with the rise of opioid dependence [10]. Studies have reported misusers describing their experiences, like “lope highs” after large consumptions of the medicine as ”better than any oxycodone” [8]. Unfortunately, high doses of loperamide have profound cardiotoxic effects, including serious dysrhythmias [1-2, 11].

## Case presentation

**Figure 1:**
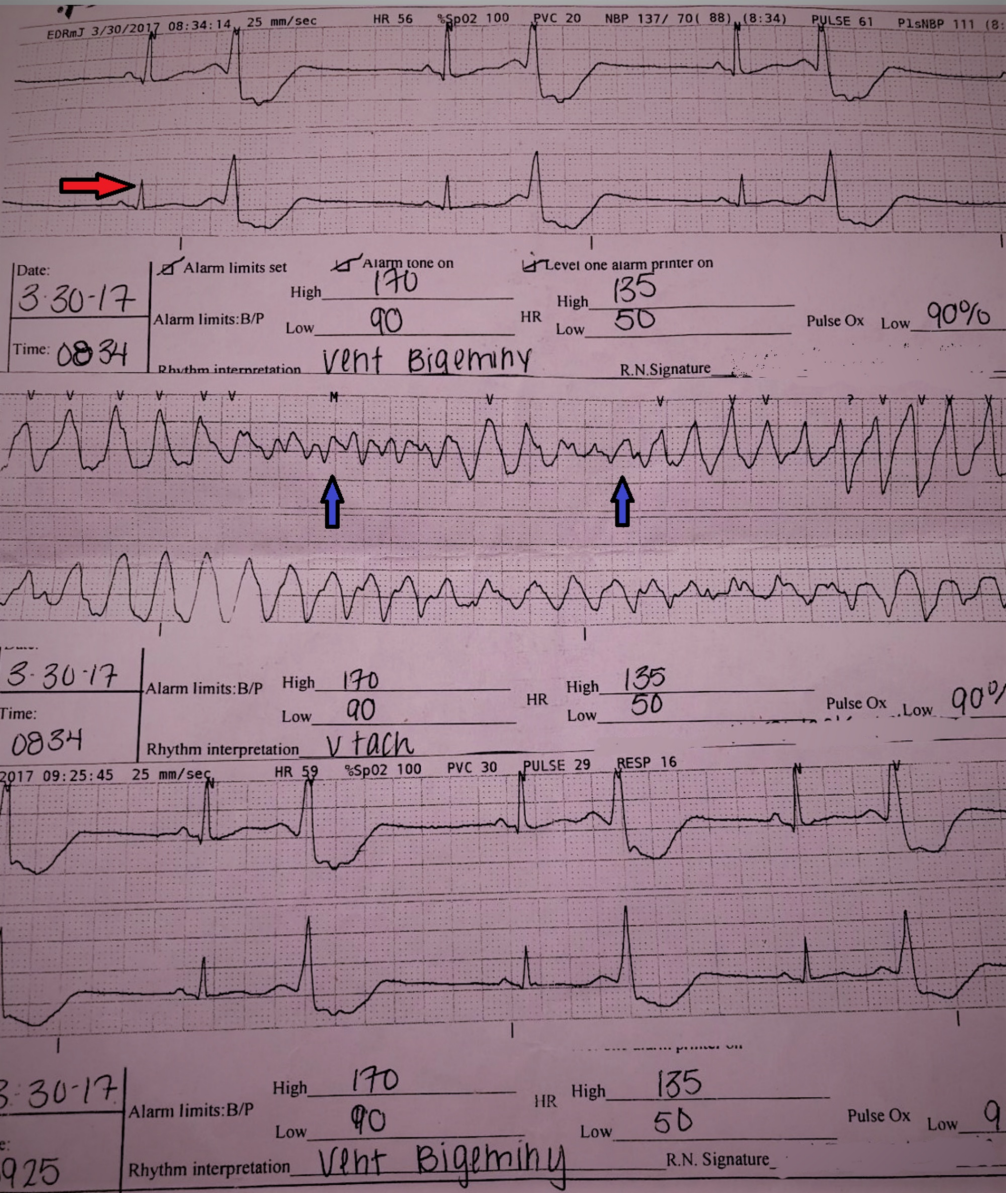
Electrocardiogram of the pateint in the ED showing polymorphic ventricular tachycardia (blue arrows) and ventricular bigeminy (red arrow).

A 32-year-old female with a past medical history of chronic back pain, fibromyalgia, chronic pain syndrome, and past opioid usage presented to our ED with atypical chest pain, palpitations, and shortness of breath. She also gave a history of near-syncopal episodes in the past. The day before she presented to the ED, the patient had a syncopal episode and "turning purple" at home, prompting her daughter to call the emergency medical services (EMS), but the patient canceled the EMS after she awakened from this episode (likely due to Torsade’s de Pointes). On arrival to the ED, she had an episode of polymorphic ventricular tachycardia, which was sustained, and the patient spontaneously self-converted to sinus rhythm with frequent premature ventricular contractions and ventricular bigeminy (Figure 1). She had a persistently prolonged QT interval, and nonspecific ST changes were noted. She was put on telemetry, where 19 beats of Torsade’s were noted. The patient denied any known personal or family history of heart disease or any congenital QT syndrome. She is an everyday cigarette smoker and denied any alcohol or any other illicit drug use. Toxicology studies were unremarkable.

**Figure 2:**
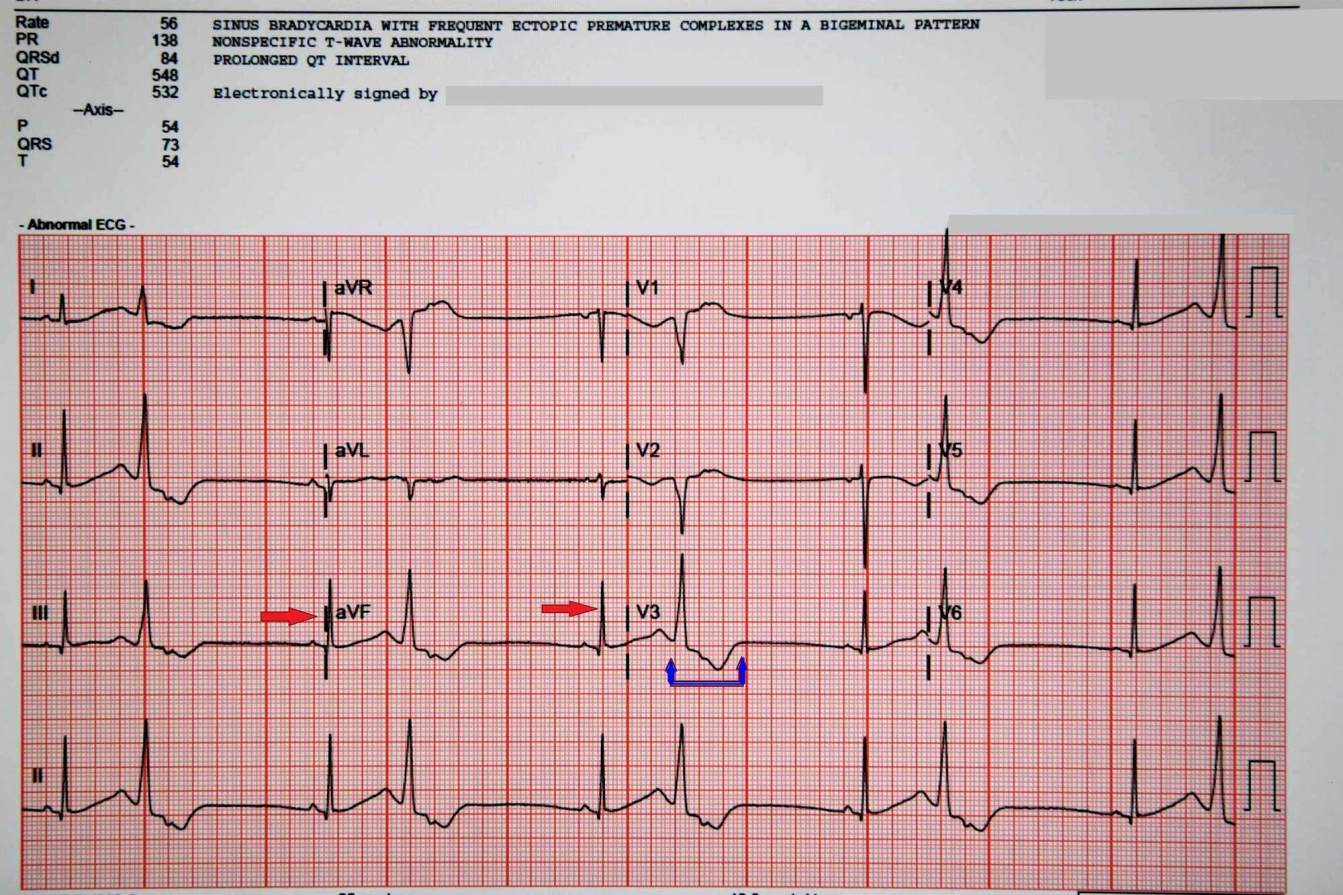
Electrocardiogram of the pateint in the ED continuing to show frequent premature ventricular contractions (red arrows) ventricular tachycardia and prolnged QT interval (blue arrows).

Labs were normal except for hypokalemia, which was replaced. Her serial cardiac enzymes were normal imaging of the chest, abdomen, and pelvis was unremarkable. The patient received amiodarone and magnesium in the ED and was put on continuous telemetry. Despite this intervention the patient continued to show ventricular bigeminy and short runs of polymorphic ventricular tachycardia (Figure 2). The patient was upgraded to the intensive care unit (ICU), for closer monitoring and evaluation. The electrophysiology and cardiology services were consulted. The patient underwent an echocardiogram, which showed a normal ejection fraction, normal right and left ventricular functions, and no valvular abnormalities. The patient later endorsed that she was on oxycodone, fentanyl patch, and hydrocodone for her chronic pain issues. Her primary care had weaned her off the medications, and she was not getting any more opioid prescriptions. She also confided that she had been consuming 200 tablets of 2 mg over-the-counter (OTC) loperamide every week to "extend her opioid analgesic prescription for her chronic pain issues" and that she had taken one high dose of 200 tablets of 2 mg all at once before today's ED visit. Due to the long half-life of loperamide, the patient was advised to continue admission to the ICU till the QT interval stabilizes. Further plan was made to send her home on life vest with outpatient follow-up for implantable cardiac defibrillator (ICD) therapy. She did not seem interested in any of the above-stated therapies and adamantly refused any outpatient or inpatient opiate rehabilitation. While all these discussions were taking place, the patient left the hospital against medical advice (AMA).

**Figure 3:**
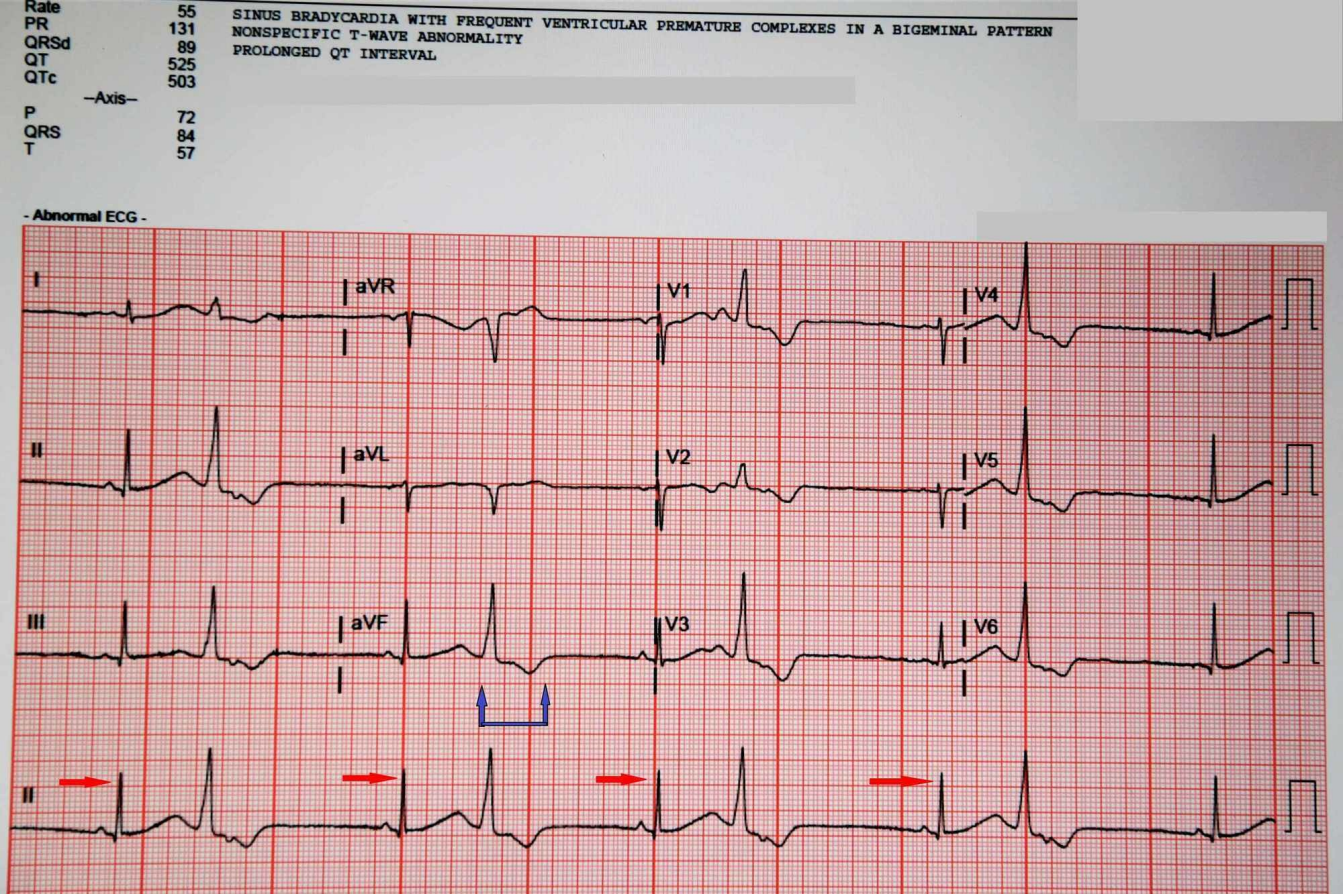
Electrocardiogram of the patient the next day when she again presented to the ED, still continuing to show the frequent premature ventricular complexes (red arrows) and bigeminal pattern along with the prolonged QT interval (blue arrows) which had only marginally improved.

The patient again presented to our ED with chest pain, shortness of breath, nausea, and vomiting on the following day. She was still bradycardic in the '50s along with persistent, prolonged QT interval and ventricular bigeminy (Figure 3). Electrophysiology and cardiology services were again consulted, and the patient also underwent coronary angiography, which did not show any significant blockage or evidence of structural heart disease. It was again reiterated to her about the importance of considering life vest versus ICD versus medical management. She was also advised to refrain from loperamide use entirely and to quit cigarette smoking. Despite all the advice and education from several physicians and hospital staff, the patient again left the hospital AMA and was unfortunately lost to follow up.

## Discussion

Loperamide is a mu-opioid receptor agonist usually used to treat diarrhea and often available as an over-the-counter medication. Unfortunately, there seems to have been a steady rise in recreational abuse for its opioid-like effects in recent years [12]. The cardiac complications have ranged from often presenting as QRS widening, QTC prolongation, ventricular tachycardia, Torsade’s de Pointes, or a Brugada-like syndrome [4, 11, 13-14]. The Illinois Poison Center (IPC) took care of 137 cases of loperamide exposures from 2014 to 2016, out of which 18 overdosed for the purpose of abuse, withdrawal prevention, or withdrawal treatment. Most loperamide abuse cases were reported to have a significantly prolonged QT interval [15]. Although the cause of the QT prolongation is not yet completely understood, the calcium channel blockage effect seen in animal models has been proposed as one of the mechanisms, among others [16]. There is a distinct structural similarity between loperamide, methadone, and the potent human ether-a-go-go (hERG) receptor blocker terbinafine, as all three of these molecules have multiple phenyl rings [17]. It is also being proposed that the QT-prolonging effect of loperamide could be due to the hERG blockade effect similar to terbinafine [16-17]. 

Loperamide has been safely used for many years without any significant problems, and the recent upsurge in its adverse effects seems to stem from the ingestion of this medication in toxic amounts. This upsurge seems to have been unfortunately propagated through the websites, web forums, and web discussions with anonymous posts. In the 2013 study on the user generated content (UGC) for the extra-medical usage of loperamide among illicit drug, Daniulaityte et al. found that almost 70% users discussed loperamide as a remedy to self-treat opioid withdrawal symptoms and 25% discussed its high doses to produce the euphoric effects [18]. Case reports from the US Food and Drug Administration Adverse Event Reporting System (FAERS) in 2016 provided evidence that toxic doses of loperamide were associated with Torsade's de Pointes and other serious cardiac events [19]. The majority of these cases occurred in the setting of drug abuse to prevent opioid withdrawal or to produce euphoric effects. Optimal management of loperamide-associated toxicity is still unclear and treatment is aimed at giving anti-arrhythmic, magnesium or sodium bicarbonate in some cases.

## Conclusions

The Food and Drug Administration (FDA) continues to undertake stringent measures after the 2014-2016 increase in deaths due to cardiac causes secondary to loperamide abuse. In June 2016, FDA issued Black Box warning about serious heart problems, including Torsade's cardiac arrest and sudden cardiac death with high doses of loperamide use. In April 2017, FDA ordered additional alert along with the Black Box warning. In January 2018, FDA started working with manufacturers to use blister packs or other single-dose packaging and to limit the number of doses per package of loperamide that could be sold OTC. More recently, in September 2019, the FDA approved the new packaging for the brand name OTC loperamide in an attempt to prevent abuse and misuse. The maximum allowed daily dose of OTC loperamide for adult usage is 8 mg/day. The new carton is to contain no more than 48 mg of loperamide and requires unit dose blister packaging.

With our case, we attempt to increase the awareness of this innocuous drug's misuse needs to spread among providers, especially primary care providers emergency physicians as well as cardiologist, who may encounter this problem more often than they previously realized. It is equally imperative to educate the patients about the potential risks of overuse. At the same time, public health awareness about this growing menace needs to be created, and more stringent laws need to be passed to prevent further life-threatening cardiac complications of loperamide's abuse.
